# A Novel Virtual Navigation Route Generation Scheme for Augmented Reality Car Navigation System

**DOI:** 10.3390/s25030820

**Published:** 2025-01-30

**Authors:** Yu-Chen Lin, Yu-Ching Chan, Ming-Chih Lin

**Affiliations:** Department of Automatic Control Engineering, Feng Chia University, Taichung City 407102, Taiwan; james397520@gmail.com (Y.-C.C.); mingchih071126@gmail.com (M.-C.L.)

**Keywords:** virtual navigation route, augmented reality, navigation system, generative adversarial network, long short-term memory network, semantic segmentation

## Abstract

This paper develops a novel virtual navigation route generation scheme for an augmented reality (AR) car navigation system based on the generative adversarial network–long short-term memory network (GAN–LSTM) framework with an integrated camera and GPS module. Unlike the present AR car navigation systems, the virtual navigation route is “autonomously” generated in captured images rather than superimposed on the image utilizing the pre-rendered 3D content, such as an arrow or trajectory, which not only provide a more authentic and correct AR effect to the user but also correctly guide the driver earlier when driving in complex road traffic environments. First, an evolved fully convolutional network architecture which uses a top-view image through an inverse perspective mapping scheme as input is utilized to obtain a more accurate semantic segmentation result for the lane markings in the traffic scene. Next, according to the above segmentation result and known location information from path planning, an AR Navigation-Nets based on an LSTM framework is proposed to predict the global relationship codes of the virtual navigation route. Simultaneously, the discriminator is utilized to evaluate the generated virtual navigation route that can approximate the real-world vehicle trajectory. Finally, the virtual navigation route can be superimposed on the original image with the correct ratio and position through an IPM process.

## 1. Introduction

**Figure 1:**
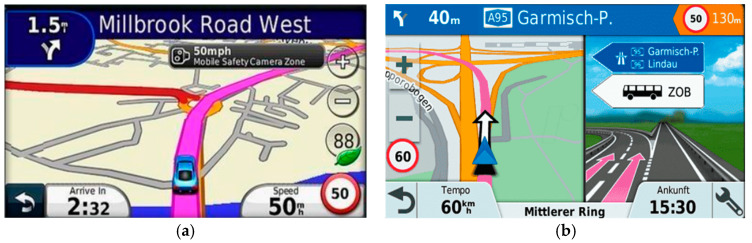
Commercial automotive navigation system (Garmin International Inc., Olathe, KS, USA). (**a**) superimposing the virtual guidance path on the electronic map and combining voice for route guidance to help drivers navigate; (**b**) navigation systems combine electronic maps and virtual reality.

Recently, the vigorous development of virtual reality and augmented reality, coupled with the rapid development of mobile communication technology in the integration of various sensors and positioning technologies, have allowed the navigation system to continue to evolve. Nowadays, most of the car navigation systems on the market are still mainly based on the method of superimposing the virtual guidance path on the electronic map and combining voice for route guidance to help drivers navigate, as shown in Figure 1a [1]. However, drivers may take a wrong turn due to the misunderstanding of displayed instructions or navigation errors when driving in complicated road conditions, such as multiple entrances for nearby intersections; this may even cause potential safety issues as well, especially for unfamiliar road conditions, long-range driving, and new drivers. According to statistical reports, visual–manual distraction due to operating a navigation system is typically associated with 5~25% [2], and even up to 80%, of all crashes, as well as significant increases in risk [3].

In addition, many navigation system companies have launched navigation systems that combine electronic maps and virtual reality, as shown in Figure 1b [4]. While providing visual guidance with virtual maps or street view images can give users a sense of being in the environment, visual guidance through 2D/3D VR modeling is often still not intuitive enough for drivers, and the projected images still differ from the real road situations when encountering complex road environments such as multi-layer roads, multi-lane intersections, multiple entrances for nearby intersections, and multi-line gates. Therefore, it is still difficult for the driver to immediately understand the road guidance given, resulting in a deviation from the route to be navigated. Moreover, constructing highly realistic 2D/3D images requires high production costs and a long production time. At present, most of the 2D/3D images are only available at highway entrances and exits, multi-lane roads, or main roads.

**Figure 2:**
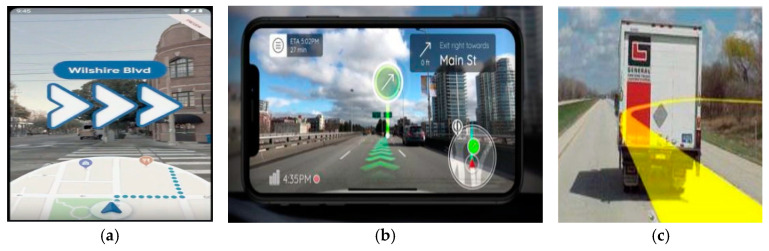
AR navigation systems. (**a**) Google’s live view [6]; (**b**) Phiar’s App [7]; and (**c**) Dr. W. Narzt [11], showing a hidden exit, where other vehicles or rises in the landscape restrict the driver’s view.

With the development of augmented reality (AR), in recent years, some companies have introduced navigation systems with augmented reality. As shown in Figure 2a, Google (Google LLC, Mountain View, California, CA, USA) launched an augmented reality navigation function in Google Maps [5] in 2018. The navigation information is superimposed on the real-life image obtained from the user’s mobile phone lens and Google image data. American system company Phiar (Phiar Technologies Inc., Redwood, California, CA, USA) has also developed a set of augmented reality car navigation applications on iOS [6], as shown in Figure 2b. Instead of displaying instructions on a digital map, the smart phone directly displays the front traffic conditions, where all navigation instructions are annotated as arrows on the live video stream to guide the driver. Although this type of AR augmented reality navigation system can indeed provide drivers with more intuitive guidance, we also found that these AR navigation systems do not consider the state of the vehicle in the context of the current road environment, so they still cannot provide the correct AR navigation effect for the user. As shown in Figure 2c below, we can observe that when there is a car ahead, the guidance icon will obscure the vehicle ahead, which will block the user from seeing the vehicle ahead, therefore, causing a collision. In addition, the current augmented reality navigation system does not consider the proportional relationship between the virtual guidance icon and the real image pixels when the virtual direction guidance icon is superimposed on the real image. If the vehicle is driving on uneven roads, the virtual direction guidance icon will point to the sky or to the ground, which is more likely to cause misjudgment by the users.

In the past decades, a lot of studies also proposed the lane-level navigation system [7,8,9,10,11,12,13] so that a navigation system can operate at the lane level by fusing the lane-level road information using camera and ground plane estimation based on geometric relationship, as well as displaying early augmented turning maneuvers and lane recommendations to inform the driver so they can respond quickly and to support safe driving practices. Most of the studies on navigation systems focused on the positioning issue, such as when the GPS error is too large or there is no GPS signal, and the lane line recognition and vehicle dynamic information are combined to confirm the current vehicle’s position in the lane to assist the vehicle’s navigation (real-time route planning), lane change, turning, etc. The most important purpose of the AR navigation system is to make the user aware of the correct direction information in real time, so as to make appropriate driving decisions. However, nowadays most of the research in AR navigation systems focuses on how to make the virtual navigation icon attach to the correct position in the image, i.e., in front of the center of the vehicle’s own lane (it focuses on the lane-level positioning and map-matching issue), rather than focusing on how to provide the correct directional guidance, especially when driving in a complex road environment. Notably, these two purposes are quite different. Hence, most AR navigation systems still cannot provide accurate directional guidance in the complex road environment, such as a complex road environment with fast and slow lanes, uneven roads, etc. For example, in Figure 2c, occlusion is the key concern, and it must be considered to avoid confusion in depth between real-world objects and AR graphics. Hence, the object detection function must be developed to infer the ordinal position in the depth of the occluded object, which will additionally present a larger computational burden. Furthermore, it is worth noting that if the user is driving in the inner lanes (fast lanes) of multi-lane roads or a complex road environment with fast and slow lanes, the current navigation systems can only provide guidance on turning, without guiding the vehicle to switch to the outer lanes in advance before getting to the intersection in which they are to turn. Such issues often cause incidences such as missing the intersections or last-minute lane changing, which could lead to serious rear collision accidents; present AR navigation systems do not consider the road environment information and the limitation of the GPS positioning errors, which makes it difficult for the current navigation systems to provide accurate directional guidance in advance when driving in complex road environment. Therefore, in recent years, AR navigation systems have not yet been commercialized or used in on-board navigation systems.

In view of this, this paper presents a virtual navigation route generation scheme based on a generative adversarial network–long short-term memory network (GAN–LSTM) framework for implementing a novel augmented reality navigation system. Unlike the present AR navigation system, the virtual navigation route is “autonomously” generated in captured images rather than superimposed on the image by utilizing the pre-rendered 3D content, such as an arrow or trajectory, and can provide a more authentic and correct AR effect for the user when driving in a complex road traffic environment. Moreover, the virtual navigation route can autonomously generate the correct position using the proposed stacked LSTMs strategy, so the continuity and contextual relevance of the virtual navigation coding data can be effectively ensured. This can effectively prevent the virtual navigation path from being superimposed onto and obstructing objects in the augmented reality, and it can also effectively improve the navigation accuracy of the augmented reality.

## 2. Related Works

The ability to sense and recognize the surrounding environment of the vehicle is one of the key technologies in this study. Since it is necessary to obtain the current lane position and types of lane markings in front of the vehicle to subsequently generate a suitable virtual navigation route, the technology for these is needed to perform such tasks. Traditional lane line identification methods are mostly based on image feature type identification methods, such as the Hough Transform [14], inverse perspective mapping (IPM) [15], and Canny edge detector [16], etc. Sukumar et al. [17] proposed an improved vision-based framework for lane detection and departure warning in ADASs, utilizing SDCT-based edge filters, RANSAC for lane model fitting, and lateral offset ratio computation for warning systems. However, traditional image recognition methods are limited by factors such as lighting, road complexity, and even harsh weather, resulting in insufficient reliability of the result.

To improve the drawbacks caused by the use of hand-crafted features, learning-based approaches have been proposed for the surrounding environment perception [18,19,20,21,22]. Itu et al. [23] proposed a lightweight CNN for predicting vehicle speed, braking, and emergency-brake events using monocular video sequences, enhancing road safety and enabling efficient deployment. Zhang et al. [24] proposed a ripple lane line detection network (RILLD-Net) based on a convolutional neural network and residual module to identify lane lines. The passes feature maps between modules at various distances that are adopted so as to learn more efficiently and the characteristics of each stage. In this study, an evolved fully convolutional network (EFCN) [25] is adopted to detect lane markings in a traffic scene. By introducing the residual network architecture of ResNet and concatenating the shallow feature map with the feature map of the corresponding scale in the up-sampling stage, it can effectively save the image details that may be lost when the scale of the feature map is reduced, which leads to better results for the semantic segmentation of lane markings.

Recently, GRU [26] and LSTM [27] have been widely adopted to deal with time series problems and improved the problems of gradient exploding and vanishing encountered when using a recurrent neural network (RNN). They can also understand the importance of different time series lengths better than ordinary RNN architecture and are able to predict longer time series, which results in better performance in forecasting. For the task of trajectory prediction, Ge et al. [28] and Zhang [29] both utilized GRU as the backbone. In various complex scenarios, their work highlighted GRU’s effectiveness in sequential trajectory modeling. Hou et al. [30] proposed a multi-target trajectory prediction model based on LSTM, which not only used the horizontal and vertical relative positions of the target vehicle, but also combined the speed, acceleration, and size of the surrounding vehicles to learn the interaction between the target vehicle and all vehicles in the scene so as to predict the long-term trajectory of the vehicles in the scene. In 2019, Sadeghian et al. proposed a generative adversarial network with an LSTM architecture (GAN–LSTM) [31], which used the image and the walking route of the pedestrian in the image to refer to the interaction between different objects to predict the possible subsequent walking route of the pedestrian in the image. Similarly, Wu et al. [32] proposed a novel trajectory generator based on a constrained GAN and a latent variables predictor to improve the accuracy and realism of pedestrian trajectory predictions. Taking the above articles as inspiration, this study leverages the ability of LSTM to process time-series data to predict the current virtual navigation route, while using GAN [33] to optimize the predicted results, making it more compatible with the actual road environment by automatically generating the most appropriate code for the virtual navigation route at present. Finally, the inverse perspective mapping (IPM) scheme is used to achieve the best augmented reality navigation effect by superimposing the generated virtual navigation route information on the original image with the correct proportions.

The main contributions of the proposed scheme are as follows:

- Unlike the present AR navigation system, the virtual navigation route is “autonomously” generated in captured images rather than superimposed on the image by utilizing the pre-rendered 3D content, such as an arrow or trajectory, and not only provides a more authentic and correct AR effect for the user but also correctly guides the driver earlier when driving in complex road traffic environments to effectively avoid dangerous driving maneuvers such as missing the exit, entering the wrong highway, and even last-minute lane changes, which can lead to serious rear collision accidents.
- In order to enhance the authenticity of the virtual navigation information superimposed on the real image, the inverse perspective mapping approach is adopted to convert the original image into a bird’s-eye-view image for road environment identification and virtual navigation route autonomous generation, which not only effectively reduced the volume of calculation, but also achieved the best augmented reality navigation effect. The virtual navigation route can be superimposed on the original image with the most correct 3D ratio and position to implement the best augmented reality navigation.
- The proposed system has been implemented in a real vehicle and the effectiveness of the proposed scheme is shown by conducting various tests of real road environments; in addition, the potential utility of the proposed invention for implementing the autonomous system or advanced drive assistance system (ADAS) in next-generation vehicles can be demonstrated and will have a significant automotive industrial impact.

## 3. System Description

**Figure 3:**
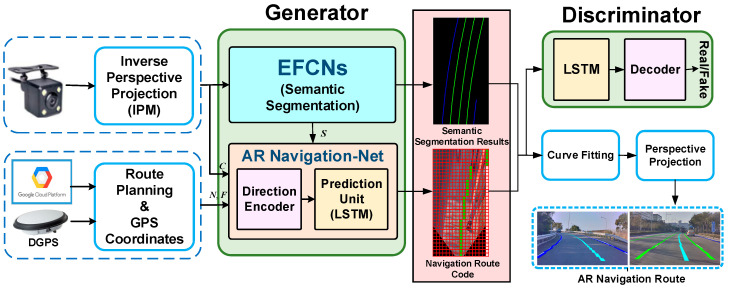
Overview of the proposed augmented reality navigation system.

The aim of this paper is to present a novel virtual navigation route generation methodology using the generative adversarial network–long short-term memory network (GAN–LSTM) framework with an integrated GPS module for implementing a novel augmented reality navigation system. Figure 3 illustrates the proposed virtual generation scheme based on GAN–LSTM architecture for a novel AR navigation system, showing that the study has three separate parts, i.e., route planning and coordinate data acquisition, dataset construction, and virtual navigation route generation.

The route planning is mainly carried out by the Google Directions API [34], and the coordinate information for all of the anchor points in the planned route is retrieved at the same time as the input for the subsequent virtual navigation route generation model. Next, a virtual navigation route autonomous generation model based on GAN–LSTM architecture is developed, where the generator is mainly composed of evolved fully convolutional networks (EFCNs) and our proposed AR Navigation-Net. The EFCNs network is mainly adopted to obtain lane markings in front of the current vehicle, including the location and category of lane lines. It is worth noting that this research uses inverse perspective mapping, IPM [15], to convert the original image into a bird’s-eye-view image and uses it for road environment identification and virtual guidance route generation in order to effectively reduce the amount of computation. Subsequently, according to the aforementioned results of the perception of the lane environment, the positioning information in the planned route, and the GPS position of the vehicle as the input of the augmented reality navigation network (AR Navigation-Net) developed in this research are combined with the long short-term memory networks (LSTM) framework to obtain the encoding result for the virtual navigation route. Simultaneously, the discriminator is used to determine the quality of the result from the generator. Through the repeated use of discriminators and generators, the generated result for the virtual navigation route can be closer to the real vehicle’s supposed travel trajectory. Consequently, a complete virtual navigation route is fitted through the quadratic curve. In order to improve the authenticity of the virtual navigation information superimposed on the real image, we finally convert the obtained virtual navigation route back to the original image through perspective mapping, so as to obtain the most realistic and correct 3D scaling ratio relationship when superimposed on the original image. Thus, the best effect for augmented reality navigation can be achieved.

### 3.1. Route Planning and Coordinate Data Acquisition

**Figure 4:**
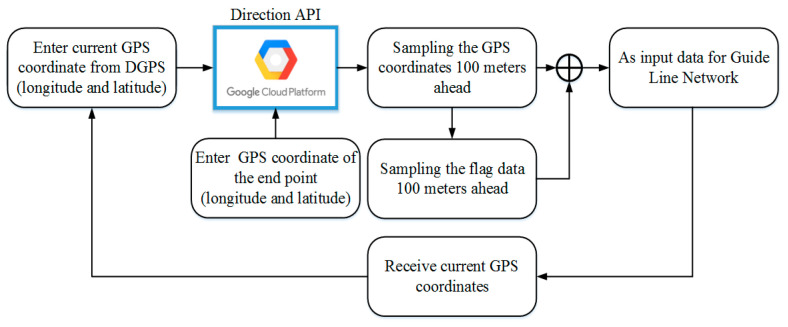
Flowchart of the route planning and coordinate data acquisition.

This research mainly uses the Directions API [34] from the Google Cloud Platform to realize path planning and coordinate information acquisition for all of the anchor points in the planned route. First, the user manually enters the destination for the Directions API to plan the best navigation route and the location data for the 100 m ahead of the current position will be retrieved as the basis for virtual navigation route autonomous generation. The flowchart of the route planning and coordinate data acquisition is shown in Figure 4.

First, the desired destination is entered by the user and then the Google Directions API is used to achieve the route planning while obtaining the latitude and longitude position information for all of the anchor points in the route. During the generation of subsequent virtual navigation routes, the current location of the vehicle and the location data for the 100 m ahead will be used as the input for the AR Navigation-Net network. In addition, it must be considered that when the vehicle is less than the last 100 m away from the end position, the latitude and longitude position for the next 100 m will have empty values, which causes errors in the subsequent generation of the virtual guidance route. Therefore, the flag information must be considered as the generated ending information of the final route position to ensure the correctness of the information when the subsequent virtual navigation route is generated.

### 3.2. Dataset Construction

**Figure 5:**
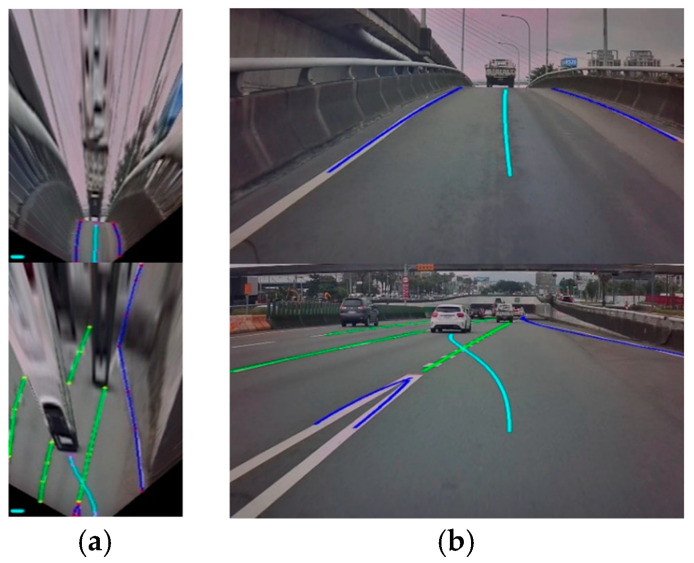
Datasets. (**a**) Dataset construction by bird’s-eye-view image. (**b**) Verification of the constructed dataset on the original perspective.

In order to superimpose the virtual navigation route on the real image with the correct ratio and position, and to effectively reduce the amount of computation, this study is mainly based on the bird’s-eye-view image to realize the proposed virtual navigation route generation algorithm. Moreover, from the original image, i.e., first-person perspective, it is difficult to efficiently and consistently construct the sample data for the virtual navigation route that conforms to the shape of the road with correct proportional relationship. Therefore, this study mainly uses inverse perspective mapping to convert the original image to a bird’s-eye view, and then performs manual sample production. The bird’s-eye-view image has the advantage of a consistent proportional relationship, which can make manual sample production more consistent and convenient. Figure 5 shows the dataset’s construction when driving in different road environments. The image datasets are built based on the top-view image by manual operation, which includes navigation path and lane marking (changeable and non-changeable lane lines), as shown in Figure 5a. The dataset results are demonstrated on the original perspective in Figure 5b. It is obvious that the blue line in Figure 5 means the entering of a forbidden zone, crossing direction separation lines or lines indicating the prohibition of lane changes, such as a barrier line, double solid lines, solid white/yellow line, and freeway entrance and exit markings. In addition, the green line indicates that the vehicle can change lanes or merge, such as the dotted white line and broken white/yellow line. The navigation path (cyan) is mainly for the markings of the appropriate navigation path through experienced and law-abiding drivers, and applies the recorded GPS information beforehand, i.e., future target position, as the basis for the direction of the navigation path. In order to make the manual sample more consistent and convenient, and at the same time in line with the actual driving conditions of real driving, this paper uses the inverse perspective mapping scheme to convert the original image to a bird’s-eye view because it has the advantage of a consistent proportional relationship, which can make manual sample production more consistent and convenient.

**Remark 1:**  *As the inverse perspective mapping is mainly based on the assumption of moving on a flat road, once the road has height variation, this assumption is lost, which thus leads to incorrect conversion results. It is worth noting, however, that although the bird’s-eye-view image generated from inverse perspective mapping may have errors, the correct navigation path and lane marking can still be drawn in the image as training data. The proposed GAN–LSTM-based model can still directly and effectively generate virtual navigation route results for highly unpredictable road conditions. Therefore, the proposed method is not limited by the hypothesis that perspective mapping is only applicable to images of flat roads. We just need to pay attention to the accuracy of the training samples when manually generating samples.*

## 4. Virtual Navigation Route Generation

In this study, a virtual navigation route generation scheme using a GAN–LSTM-based framework with integrated GPS module is proposed to implement a novel augmented reality navigation system. A more detailed description for our proposed virtual navigation route generation scheme follows.

### 4.1. Generator

**Figure 6:**
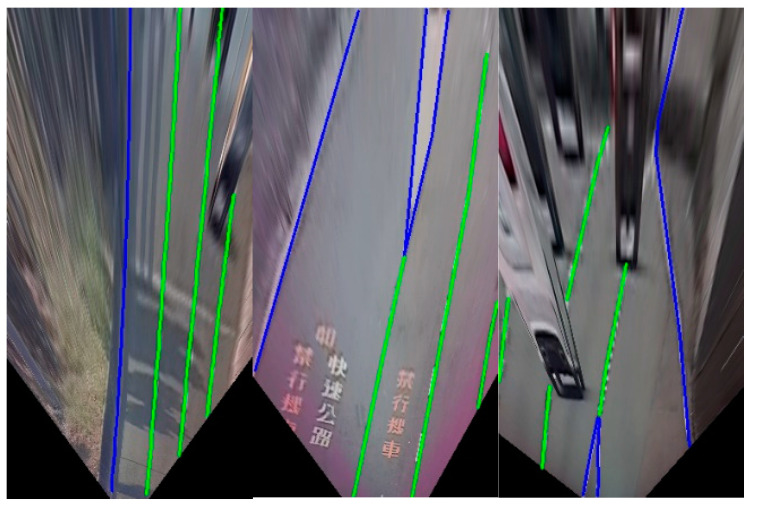
Results for lane marking detection.

The generator includes two parts: one is the evolved fully convolutional networks (EFCNs) architecture [25] for lane marking detection in traffic scenes, and the other is an AR Navigation-Net for the virtual navigation route generation task. First, EFCNs architecture is adopted to obtain a more accurate semantic segmentation result for lane markings from the top-view original image I using an inverse perspective mapping (IPM) scheme for the traffic scene. Hence, the semantic segmentation result for lane markings S and the encoding of the top-view image C are derived, respectively. In this study, the input to EFCNs is an RGB image of size 256 × 512 × 3 (width W, height H, and channels $C_{h}$, respectively). This means all images in the training set and all test images need to be of size 256 × 512 × 3. As shown in Figure 6, the lane markings under multiple complex traffic road scenes, occlusions, and sharp curves using EFCNs can not only be derived but their categories can also be obtained.

It is obvious that the blue line means entering a forbidden zone, crossing direction separation lines, or lines indicating the prohibition of lane changes, such as a barrier line, double solid lines, solid white/yellow line, and freeway entrance and exit markings. In addition, the green line indicates that the vehicle can change lanes or merge, such as a dotted white line and broken white/yellow line. It is noticed that the GAN training strategy is introduced to effectively obtain the correct and complete lane marking information, including the lane marking positions and lane types, thereby improving the accuracy of subsequent virtual navigation route generation.

**Remark 2:**  *In this paper, the semantic segmentation results for the road environment, such as the lane marking position and type, are important information that can not only be used as the basis for generating the subsequent virtual navigation route with accuracy and good consistency of the virtual navigation route, but also can be used to determine whether the navigation route instruction for changing lanes can be provided in the current road environment. For example, when a vehicle is driving in a multi-lane environment with white/yellow double solid lines, the navigation route will not generate a lane-changing guidance strategy.*

**Figure 7:**
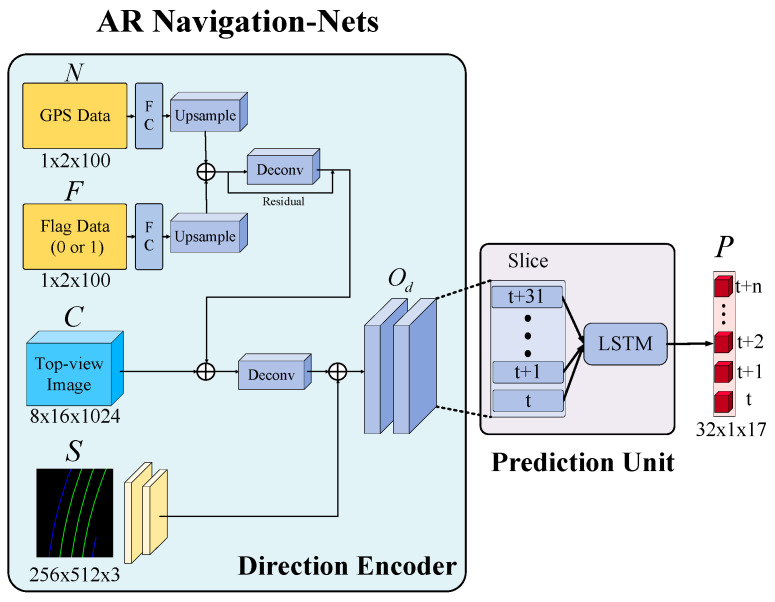
The diagram of the proposed AR Navigation-Nets.

Next, an AR Navigation-Nets based on the LSTM framework is proposed to predict the global relationship codes of virtual navigation routes; the diagram of the proposed AR Navigation-Nets is illustrated in Figure 7. The proposed AR Navigation-Nets consists of two parts: a direction encoder and a prediction unit. First, the direction encoder is mainly used to efficiently compress and encode data, and then, it learns. The data include the semantic segmentation result S, the location data N, and flag data F of 100 m in front of the vehicle after path planning, as well as feature space encoding C for the current scene. By concatenating the above feature encoding, the output of the direction encoder $O_{d}$ can then be obtained.

**Remark 3:**  *The main purpose of considering the encoding information for the current image in the direction encoder is to make the final predicted virtual navigation route conform to the real-world road environment. In addition, the semantic segmentation results of the road environment, such as the lane marking position and type, not only can be used as the basis for generating the virtual navigation route but also determine whether the navigation route instruction for changing lanes can be provided in the current road environment. For example, when a vehicle is driving in a multi-lane environment with white/yellow double solid lines, the navigation route will not generate a lane-changing guidance strategy*.

**Figure 8:**
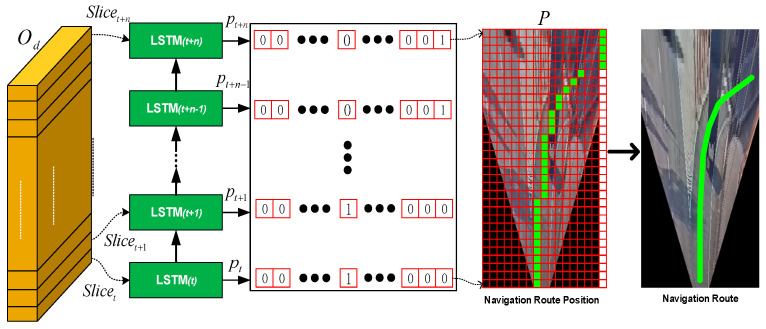
Navigation route position grid-based prediction with classification on a stacked LSTM architecture.

Subsequently, the prediction unit based on a stacked LSTM architecture is presented to the predicted position of the navigation route at t=0 to t=n in the future, for which the procedure can be regarded as a type of multi-class classification task. Figure 8 shows the navigation route position grid-based prediction with classification on a stacked LSTM architecture. First, the direction encoder output $O_{d}$ is divided into multiple slices as the sequence input for a stacked LSTM architecture. Each LSTM module is mainly used to predict the position of the navigation route in the image at each time step. The probability of the future navigation route passing through each of the grids can be calculated, setting the grid that has the highest probability calculated in each row of the navigation characteristic image as a first default value. In other words, at each time t, a probability distribution of the navigation route is distributed across all jth columns of each row in the matrix. The matrix implies correspondence to an image grid P overlay of a bird’s-eye view, and the time series forecasting of the position of the navigation route, as shown in Figure 8. Therefore, the image grid P can also be called the navigation characteristic image, in which the size of P is $\left(n+1\right)\times j$ and the n-th row is chronologically encoded with j columns representing angles around the vehicle and assembled into a grid matrix. Note that P is the set of time series forecasting results for each corresponding LSTM’s output, $P={\left[\begin{array}{cccc} p_{t} & p_{t+1} & \cdot \cdot \cdot & p_{t+n} \end{array}\right]}^{T}$, based on previous, sequential data, where the element $p_{t}$ can be represented as

(1) $$p_{t}=\mathrm{LSTM}(Slice_{t},\;h_{t};\;W_{p})$$

where $W_{p}$ is the learnable parameter of the LSTM layer, and $h_{t}$ denotes the hidden state of LSTM at time t.

In this study, the size of the grid matrix is defined as 32 × 17, where n is 31, which refers to the predicted position of the virtual navigation route at the next 32 time-points, and j is 17, with j=1~16 representing the probability of each row of the grid being the navigation route position at each time-point t, and then the grid with the highest probability grid is selected as the position for the virtual navigation route at time t, as indicated by the green grid in Figure 8. In addition, j=17 is a flag indicating whether there is a virtual navigation route location in the column. For instance, when there is an object in front of the vehicle, or the navigation has reached the edge of the screen, the 17th grid of the column will be marked by a green grid, indicating that there will be no virtual navigation coding results in this column. This can effectively prevent the virtual navigation path from being superimposed onto and obstructing objects in the augmented reality, and it can also effectively improve the navigation accuracy of the augmented reality.

**Remark 4:**  *As shown in Figure 8, 32 encodings with specific sizes can be predicted using the stacked LSTMs, effectively ensuring the continuity and contextual relevance of the virtual navigation coding data. Therefore, the proposed model will be able to realize the classification of 17 categories at each time step. Among them, the categories 1–16 refer to the probability of inferring the most likely virtual navigation route position, which is represented by the grid from left to right in Figure 8. When the 17^th^ category has been inferred, it refers to the 17^th^ grid, which implies that the position of the navigation route is not outputted at that point in time.*

**Remark 5:**  *Note that the values of* n *and* j *mainly indicate the grid fineness, which means that a larger* n *points out longer predictable time intervals, and a longer navigation route in the future to be predicted. On the other hand, a larger* j *ultimately indicates the predicted position of a navigation route can be more precise, with more smoothly generated virtual navigation instruction. However, the larger values also affect the computation time. Hence, determining the appropriate value becomes more important.*

### 4.2. Discriminator

**Figure 9:**
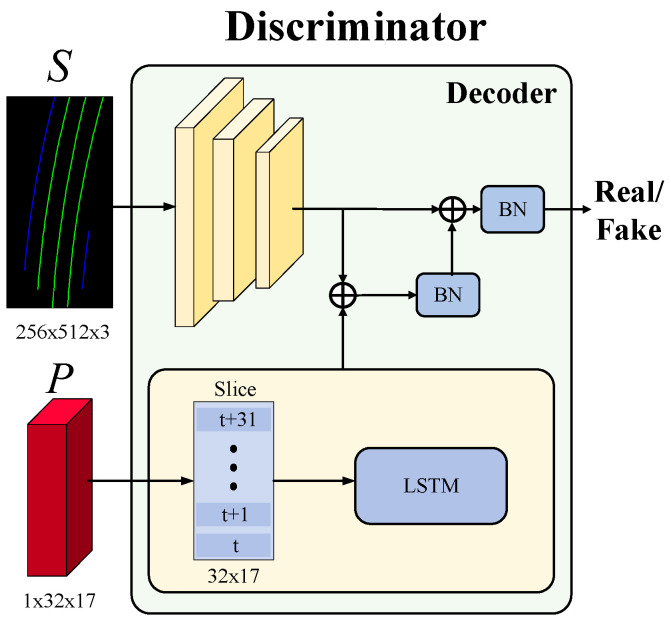
The diagram of the proposed discriminator.

In order to make the generated code for the virtual navigation route more in line with the actual road environment and the driving trajectory under real driving behavior, this paper adopts a generative adversarial training strategy, where the generator and discriminator work in opposition to each other. The confrontation between the two results in better learning for the generator. Thus, we can continuously repeat the confrontation training between the generator and the discriminator, and finally, enable the generator to generate the most appropriate lane marking semantic segmentation result and virtual navigation route according to different road situations while creating the final virtual navigation route that is closer to the trajectory driven by real driving behavior. The diagram of the discriminator is shown in Figure 9.

The total loss function based on cross entropy [35] in GAN training can be defined as

(2) $$L_{Total}=L_{S}+L_{P}+\lambda L_{GAN}$$

where

(3) $$\begin{array}{l} L_{S}=\sum_{i=1}^{m}H_{S}\left(G\left(X_{i}\right),Y_{i}^{S}\right),\;L_{P}=\sum_{i=1}^{m}H_{P}\left(G\left(X_{i}\right),Y_{i}^{P}\right),\\ L_{GAN}=\sum_{i=1}^{m}\left\{H_{G}\left(D\left(Y_{i}\right),\;1\right)+H_{G}\left(D\left(G(X_{i}),0\right)\right)\right\} \end{array}$$

where $L_{S}$ and $L_{P}$ are the loss functions of semantic segmentation s and navigation route code P, respectively. $L_{GAN}$ is a GAN loss function. The weight λ>0 is a hyperparameter which controls the contribution of each loss component and is determined experimentally. $G(X_{i})$ is the probability of event $X_{i}$ estimated by the generator from the i-th training set. $X_{i}\in \{I_{i},\;N_{i},\;F_{i}\}$, in which $I_{i},\;N_{i},\;F_{i}$ are the top-view original image, coordinate data by route planning and flag data, respectively. $Y_{i}=\left\{Y_{i}^{S},\;Y_{i}^{P}\right\}$ are the training datasets, where $Y_{i}^{S}$ and $Y_{i}^{P}$ are the lane marker dataset and navigation route dataset based on the top-view image, respectively; please refer to Figure 5a. $H_{S}$, $H_{P}$, and $H_{G}$ are the cross entropy functions of semantic segmentation S, navigation route code P, and GAN, respectively, which can be expressed as

(4) $$\begin{array}{l} H_{S}\left({\tilde{Y}}^{S},\;Y^{S}\right)=-\sum^{W\times H}\sum^{C_{h}}Y^{S}\ln{\tilde{Y}}^{S},\\ H_{P}\left({\tilde{Y}}^{P},\;Y^{P}\right)=-\sum^{n+1}\sum^{j}Y^{P}\ln{\tilde{Y}}^{P},\\ H_{G}\left({\tilde{Y}}^{G},\;Y^{G}\right)=-Y^{G}\ln\left({\tilde{Y}}^{G}\right)+\left(1-Y^{G}\right)\ln\left(1-{\tilde{Y}}^{G}\right) \end{array}$$

where W×H is the size of RGB images; $C_{h}$ are the channels of RGB images; $\left(n+1\right)\times j$ is the size of the grid matrix; ${\tilde{Y}}^{G}$ is the output of the discriminator; and $Y^{G}=\left\{0,\;1\right\}$ is the label of the real and fake results.

## 5. Experimental Results

We demonstrated our approach for performing GAN–LSTM-based navigation route autonomous generation for different road scenarios in an adversarial training and then the display of the AR navigation route on the screen. In addition, to validate the performance and effectiveness of the developed virtual navigation route autonomous generation scheme, some of the complex traffic scenes, such as multi-lane intersections, multiple entrances for nearby intersections ahead, and multi-line gates, etc., were considered. Finally, a quantitative comparison of adversarial learning and non-adversarial learning strategies for the generator was performed, including the results for lane markings and virtual navigation route coding. The experimental results provide credible evidence that proves the favorable contributions of the proposed approach. The experimental results of the numerous traffic scenario challenges for our developed AR navigation system can be visualized via videos which can be found at the following link: https://www.youtube.com/watch?v=BKCFgWobVc4 (accessed on 16 January 2025).

### 5.1. Demonstration of Virtual Navigation Route Autonomous Generation

**Figure 10:**
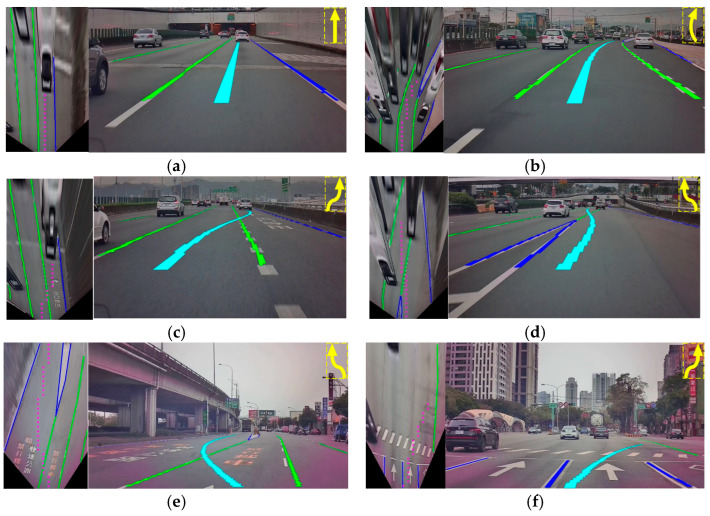
Demonstration of the proposed AR navigation system in numerous challenging traffic scenes (including lane marking detection and virtual navigation route autonomous generation results). (**a**) Multi-lane road scenario on straight road. (**b**) Multi-lane road scenario on curved road. (**c**) Lane change to exit lane guidance on approaching the highway exit. (**d**) Merging onto highway acceleration lane guidance. (**e**) Entering a freeway on-ramp guidance. (**f**) Fast to slow lane change early guidance on approaching a right turn at the next intersection.

The EFCNs were adopted to derive the semantic segmentation result for the lane markings via a top-view image using an IPM scheme. Figure 10 shows the lane marking detection results under multiple complex traffic road scenes. The blue lines indicate a white/yellow line and a barrier line in a freeway entrance and exit, which means the vehicles are prohibited from moving in either direction or crossing the lines or the outside edge of each lane. The green lines indicate the dotted white line and broken white/yellow line, over which the vehicle can change lanes or merge.

Input data for developing AR Navigation-Net included the segmentation result for lane markings, the location point information for the ego vehicle’s next 100 m of the navigation route, the flagging data, and the encoding of the current image. The navigation characteristic image is obtained through concatenation and coding, and the virtual navigation route is predicted with the long short-term memory (LSTM) networks. In addition, with GAN training, the encoding of the result can be closer to the actual road environment and the driving trajectory under real driving behavior to generate the most appropriate virtual navigation route coding result. The pink dots in the left picture of each example in Figure 10 are the coding prediction results of the virtual navigation route using LSTM. Next, the quadratic function is adopted to perform curve fitting so that the virtual navigation route under the top-view image can be obtained. Finally, the virtual navigation route is converted back to the original image by the IPM approach to achieve the augmented reality effect. Several challenging road scenarios are considered to validate the proposed AR navigation system, for which the experimental results are shown in Figure 10, where the light blue line is the virtual navigation route. In addition, Figure 10 also displays the current navigation information, including the current vehicle position (red dot) and navigation information, in the upper right corner of each figure in a map.

Figure 10a,b show the proposed AR navigation system in a multi-lane road environment, from which it is obvious that the virtual navigation route can not only correspond to the current road curvature but also effectively present the correct proportional relationship to achieve an effective augmented effect. Figure 10c shows the virtual navigation route for approaching a highway exit. The proposed approach can automatically generate a virtual navigation route that changes to the exit lane in advance. In addition, we observe that the end of the generated virtual navigation route stops at the rear of the vehicle in front, which effectively provides the correct augmented reality effect; simultaneously, it allows the driver to maintain their sense of space. Figure 10d is the virtual navigation route for merging onto the freeway from the on-ramp. Note that the virtual navigation route for changing lanes is mainly based on the identification result of the lane markings. If the current markings are the double white lines or channelizing line marking, i.e., it is illegal to change lanes, as shown in the blue marking in Figure 10d, the virtual navigation route with lane changing will not be generated. Figure 10e shows the guiding of the vehicle onto the entry ramp of the expressway for the generation of the virtual navigation route instruction for changing lanes to the entry ramp. Figure 10f shows a multi-lane complex urban road environment with a divisional island. According to the navigation information on the upper right, the vehicle wants to turn right at the next intersection ahead. The developed approach can guide the vehicle to the outermost lane (slow lane) at the intersection before the turn so that the vehicle can immediately turn right at the next intersection. This result effectively resolves the problem with the current navigation systems not being able to provide effective navigation in complex road environments or when the driver is unfamiliar with the road environment, which can consequently cause dangerous behaviors such as missing the exit or making last-minute lane changes. Moreover, it is obvious that the autonomously generated virtual navigation route can not only correspond to the current road curvature but also effectively present the correct proportional relationship to achieve the best augmented reality navigation effect.

**Remark 6:**  *Due to the limitation of GPS positioning errors, the traditional navigation systems are not able to precisely locate the vehicle in complex multi-lane environments separated by traffic islands as shown in Figure 10f. As a result, they cannot guide the vehicle to switch from fast lanes into slow lanes in advance before making a turn at the intersection, which often causes drivers to miss the exit or make multiple last-minute lane changes, which could cause serious rear collisions on highways, especially for first-time drivers or drivers who are not familiar with the area because they are too focused on the navigation system. Therefore, this study uses the lane marking recognition results, the GPS positioning information of the vehicle, and the upcoming navigation information from AR Navigation-Nets developed based on GAN–LSTM to autonomously determine and automatically generate a virtual navigation path that guides the vehicle to change to the outermost lane in the intersection before the turn so as to provide the driver with correct turning instruction at the next intersection.*

In this paper, a car PC with Intel i7-4770 @3.4Ghz CPU, NVIDIA RTX3080 GPU was used as a computing platform to implement the proposed algorithms; in addition, we have implemented this on a real vehicle, and the execution speed was 15 FPS (0.067 s per frame). For navigation applications, the computation speed can satisfy all vehicle speeds sufficiently. For example, if driving at 100 km/hr., the vehicle will travel 27.78 m per second; our system outputs once every 0.067 s, which can be converted into a vehicle driving 1.86 m forward. The proposed AR navigation system mainly presents the navigation information to the driver 100 m in the future; therefore, the developed approach is real-time for AR navigation applications, and more than sufficient. In the future, we will focus on the implementation of a real-time automotive embedded system for the proposed approach.

### 5.2. Evaluating the Effect of Adversarial Training

In this paper, the generative adversarial learning strategy is mainly utilized for model training to obtain a virtual navigation route closer to the real current lane conditions and driver’s behavior by using competition between the generator and discriminator for both networks to learn simultaneously. In this study, the RMSprop and Adam optimizers were used to update generator and discriminator parameters with the initial learning rates of $1\times 10^{-4}$ and $1\times 10^{-3}$, respectively. In the training process, in addition to comparing the results from the generator with the ground truth, a discriminator was added to determine the probability of error when the current generator’s prediction result was that of training sample data. Therefore, the error between the calculation of the generated result and the training sample data was used as the direction of training; by the time the generator was able to generate a certain level of results, the influence of the discriminator was gradually enlarged, so that the direction of gradient update was less susceptible to interference from the discriminator during the training of the generator. Thus, the weight λ from (2) will gradually increase with the amount of training. In other words, after the generator has the ability to generate a certain level of results, the discriminator’s error will be used as a reference. The quantitative comparison between the adversarial and non-adversarial training strategy for the generator, including the results for lane marking semantic segmentation and virtual navigation route encoding, are presented in Table 1. 

**Figure 11:**
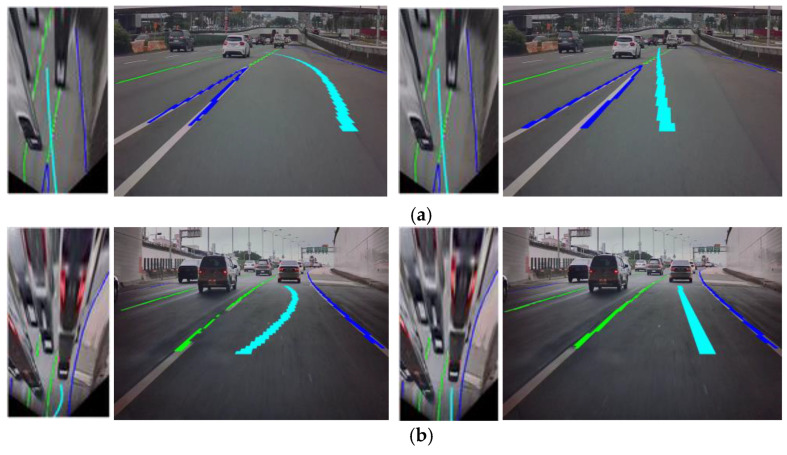
Comparison of two different training strategies for the generator. (**a**) Non-adversarial training. (**b**) Adversarial training.

It is obvious that in terms of both the pixel accuracy (PA) and the mean pixel accuracy (MPA), the results of the generator through adversarial learning are better than those of non-adversarial learning. The experimental results for lane marking recognition and virtual navigation route for the two different training strategies are demonstrated in Figure 11.

**Note 1:**  *Regarding the evaluation metrics in semantic segmentation tasks, the pixel accuracy (PA) metric denotes the percentage of pixels that are accurately classified in the image. Since there are usually multiple classes present in the semantic segmentation task, the mean pixel accuracy (MPA) represents the class average accuracy.*

In the first case, i.e., the navigation scene where the vehicle merges into the main lane, the virtual navigation route results obtained by the two training strategies seem to achieve correct and good directional guidance. However, from observing the virtual navigation route generated in the top view, it can be seen that the path from Figure 11a guides the vehicle to the right side of the current lane first before guiding the vehicle quickly to the main lane from the back end, which is less in line with the driving trajectory in real-world circumstances. On the contrary, the path from adversarial learning in Figure 11b leads the vehicle slowly and slightly towards the lane on the left and guides the vehicle to merge into the main lane. Therefore, the virtual navigation path generated by adversarial learning can more effectively emulate the driving trajectories in real-world circumstances. In the second scenario of going straight uphill, the virtual navigation route generated by non-adversarial learning yielded an incorrect result, as shown in Figure 11a. In addition, compared with adversarial learning, the lane marking semantic segmentation results of non-adversarial learning are also poor, i.e., more fragmented. In view of this, adversarial learning can not only achieve better semantic segmentation results for lane markings, but the virtual navigation route can also autonomously generate more realistic driving trajectories.

**Remark 7:**  *In this paper, the adversarial training and non-adversarial training used the same model. The difference is only the training strategy: one is non-adversarial training (without GAN), and the other is adversarial training (with GAN). The virtual navigation route generation in both learning strategies is based on the latitude and longitude position information for all of the anchor points in the planned route in advance, perception results of road environment, and the current GPS position of the ego vehicle, simultaneously. Hence, it can only be said that the direction of the generated route of both learning strategies has the same trend. In the adversarial training process, in addition to comparing the results from the generator with the ground truth, a discriminator is added to determine the probability of error when the current generator’s prediction result is that of the training sample data. Therefore, after the generator is able to generate a certain level of results, the discriminator’s error will be used as a reference, and penalize the generator if the mode is collapsing. By repeatedly training the discriminator and generators, the autonomously generated result of the virtual navigation route can be closer to the real vehicle travel trajectory.*

## 6. Conclusions

In this paper, a virtual navigation route autonomous generation scheme based on the generative adversarial network–long short-term memory network (GAN–LSTM) framework is proposed for implementing a novel AR navigation system. It is noticed that the virtual navigation route is “autonomously” generated from the captured image in front for superimposing on the image, rather than from pre-rendered 3D content, as in current AR navigation systems. The proposed AR system can not only provide more intuitive AR navigation information but also achieve safety assistance in front and collision avoidance. In addition, it can correctly guide the driver to change lanes earlier on undulating roads and in complex road environments in order to avoid missing the exit, going on the wrong on/off-ramp, or engaging in dangerous driving behaviors. The proposed AR navigation system was implemented in a real car and the system’s functions were verified in several complex road traffic environments. In the future, we will continue to build up the sample data under different weather and ambient brightness conditions to satisfy the robustness requirements for all weather conditions. We believe that our innovation not only holds great promise for solving a variety of practical problems and challenges, with significant market potential for the AR navigation system, but that the proposed techniques will also further benefit the advanced driver assistance systems (ADASs) and automated driving industry.

## Figures and Tables

**Table 1:** Quantitative comparison of the adversarial and non-adversarial training strategy for the generator.

Generator	Index	Non-Adversarial	Adversarial
Lane markings	PA	99.13%	99.18%
	MPA	86.53%	87.45%
Virtual navigation route	PA	99.40%	99.41%
	MPA	97.30%	97.37%

## Data Availability

Data are contained within the article.
